# Immediate versus delayed loading of implants in posterior mandible: A CBCT-based one-year comparative study

**DOI:** 10.6026/973206300214746

**Published:** 2025-12-15

**Authors:** Manpreet Kaur, Sachin Yadav, Diksha Verma, Chiluveru Manoj Kumar, Samir Mansuri, Jay Taank, Rahul Tiwari, Anil Managutti

**Affiliations:** 1 Christian Dental College and Hospital, Ludhiana, Punjab, India; 2 Department of Dentistry, Government Medical College, Alwar, Rajasthan, India; 3 Guru Nanak Dev Dental College and Research Institute, Sunam, Punjab, India; 4 Department of Oral and Maxillofacial Surgery, Sri Sai College of Dental Surgery, Vikarabad, Telangana, India; 5 Consultant Oral and Maxillofacial Surgeon, Ahmedabad, Gujarat, India; 6 Department of Oral & Maxillofacial Surgery, Chhattisgarh Dental College and Research Institute, Rajnandgaon, Chhattisgarh, India; 7 Department of Oral and Maxillofacial Surgery, Narsinhbhai Patel Dental College and Hospital, Sankalchand Patel University, Visnagar, Gujarat, India

**Keywords:** Dental implants, immediate loading, delayed loading, mandible, posterior, cone-beam computed tomography

## Abstract

Recent studies emphasize that bone remodeling within the first year is a critical determinant of long-term implant success and marginal bone loss > 1.5 mm may jeopardize prognosis. Therefore, it is of interest to assess immediate versus delayed loading of implants in the posterior mandible over one year using CBCT evaluation. Hence, forty patients were treated with single implants allocated to immediate or delayed loading groups. Outcomes included implant survival, marginal bone changes and peri-implant soft-tissue health. Both groups achieved 100% survival. CBCT revealed slightly higher bone remodeling in immediate loading, though within clinically acceptable limits. Clinical parameters were comparable. Immediate loading, under strict case selection, may thus be a viable treatment option in posterior mandible.

## Background:

Implant therapy in the posterior mandible is clinically demanding due to higher occlusal forces, dense cortical bone and anatomical considerations such as proximity to the inferior alveolar canal. Conventional delayed loading protocols, recommending 3-6 months of osseointegration before prosthetic function, were historically considered the gold standard for predictable outcomes [1]. However, patients increasingly seek reduced treatment time and earlier function, driving interest in immediate loading protocols even in high-load posterior sites [2]. The feasibility of immediate loading relies on achieving sufficient primary stability, generally defined by insertion torque ≥ 35 N.cm or implant stability quotient (ISQ) > 65 [3]. Innovations in implant macro-design, roughened surface technology and computer-guided surgery have improved the predictability of achieving such stability in posterior mandible [4]. Randomized controlled trials and meta-analyses suggest no significant difference in survival between immediate and delayed loading protocols across various jaw sites, though evidence specific to posterior mandible remains limited [5]. CBCT, with its three-dimensional imaging capability, allows accurate assessment of peri-implant bone remodeling and provides more precise evaluation than conventional periapical radiographs [6]. Recent studies emphasize that bone remodeling within the first year is a critical determinant of long-term implant success and marginal bone loss > 1.5 mm may jeopardize prognosis [7]. Therefore, it is of interest to report a one-year CBCT-based comparison of immediate versus delayed loading of implants placed in the posterior mandible.

## Materials and Methods:

This prospective controlled clinical study was conducted at a university implant center after ethical clearance and written informed consent. Forty partially edentulous patients (25-65 years; ASA I-II; non-smokers or light smokers <5/day) requiring a single implant in posterior mandible were included. Patients with uncontrolled systemic disease, parafunction, or inadequate bone volume were excluded. Patients were allocated into two equal groups: Group A (Immediate Loading)-provisional screw-retained crown delivered within 48 h of implant placement; Group B (Delayed Loading)-implant left unloaded and prosthesis delivered at 3 months. All implants were placed via flap approach with CBCT-guided planning. Only implants achieving insertion torque ≥ 35 N.cm and ISQ ≥ 65 were included for immediate loading. Final prosthesis was delivered at 3 months in both groups. Primary outcome was marginal bone change, measured mesially and distally on standardized CBCT scans at baseline and 12 months. Secondary outcomes included implant survival, probing depth (PD), bleeding on probing (BOP) and mobility. Statistical analysis was performed using paired t-tests for intragroup comparisons and Mann-Whitney U test for intergroup differences, with significance set at p < 0.05.

## Results:

## Radiographic CBCT analysis:

All 40 implants were followed up to 12 months, with no dropouts. At the mesial sites, both immediate and delayed loading groups demonstrated significant marginal bone remodeling over the one-year period. The immediate loading group showed a mean bone change of 0.52 ± 0.18 mm, while the delayed loading group presented with 0.45 ± 0.16 mm. Although both changes were statistically significant compared with baseline, intergroup analysis revealed a borderline difference (p = 0.042), indicating slightly greater mesial bone resorption in the immediate loading group without compromising clinical success (Table 1 - see PDF). At the distal sites, CBCT evaluation also revealed significant bone remodeling from baseline to 12 months in both groups. The immediate loading group exhibited a mean marginal bone change of 0.55 ± 0.20 mm, compared with 0.48 ± 0.17 mm in the delayed loading group. While both values were within acceptable clinical thresholds, the difference approached statistical significance (p = 0.051) (Table 2 - see PDF). This trend suggests marginally higher remodeling in the immediate group, consistent with early functional loading dynamics in posterior mandible.

## Discussion:

This one-year CBCT-based study compared immediate and delayed loading of implants in the posterior mandible. The survival rate of 100% in both groups aligns with earlier randomized controlled trials and systematic reviews reporting excellent short-term outcomes for both protocols [8, 9]. The mean marginal bone loss in both groups (< 0.6 mm) remained well below the critical threshold of 1.5 mm during the first year, confirming clinical success [10]. However, the immediate loading group exhibited slightly higher remodeling, with borderline significance at mesial sites. This modest increase is consistent with other CBCT and radiographic studies suggesting greater remodeling during early functional loading, though the difference is often clinically negligible [11]. Peri-implant soft-tissue parameters were similar across groups, indicating that immediate loading did not compromise soft-tissue integration or induce peri-implantitis risk during the first year. This reinforces the concept that case selection, especially ensuring high primary stability and careful occlusal adjustment, is key to immediate loading success [12]. The strength of this study is the CBCT-based evaluation, which provides a three-dimensional assessment of bone remodeling. Unlike periapical radiographs, CBCT enables precise detection of subtle volumetric changes, thus enhancing reliability of marginal bone assessment [13]. Limitations include modest sample size, single-center design and limited follow-up of 12 months. Moreover, only patients achieving high primary stability were included, potentially limiting generalizability to cases with poor bone quality. Future multicenter randomized trials with larger cohorts and longer follow-up are required to confirm whether early bone remodeling differences persist or stabilize over time [14, 15]. Overall, this study supports immediate loading as a clinically acceptable option for posterior mandible, provided strict case selection criteria are applied. Clinicians should remain vigilant about occlusal load management and regular monitoring.

## Conclusion:

Immediate and delayed loading of posterior mandibular implants demonstrated equivalent survival rates and favorable peri-implant tissue responses. Although the immediate loading group showed slightly greater marginal bone remodeling than the delayed group, the differences remained within clinically acceptable limits. These findings suggest that with proper case selection and primary stability, immediate loading is a predictable alternative. Long-term multicenter trials are recommended to validate and extend these results.
